# Glomulin and cerebral cavernous malformations 2 protein-like mutations in an extensive blaschkoid glomuvenous malformation with polydactyly

**DOI:** 10.1016/j.jdcr.2025.06.035

**Published:** 2025-07-09

**Authors:** Andrea Roso-Mares, Isabel Andújar Perez, David O. Schairer, Bryan K. Sun

**Affiliations:** a Department of Dermatology, University of California San Diego, San Diego, California; b Faculty of Medicine, Department of Pharmacology, University of Valencia, Valencia, Spain; c Division of Pediatric and Adolescent Dermatology, Rady Children’s Hospital San Diego, San Diego, California; d Department of Dermatology, University of California, Irvine, Irvine, California

**Keywords:** CCM2L, genetics, glomuvenous malformation, skin

## Introduction

Glomuvenous malformations (GVMs) are vascular anomalies comprised of glomus cells lining dilated venous channels.^1^ Clinically, they appear as blue-purple patches, plaques, or nodules that can thicken with time, causing potential disfigurement and pain. GVMs are associated with autosomal dominant, loss-of-function mutations in the *glomulin* (*GLMN*) gene, following a 2-hit hypothesis in which a somatic second-hit mutation triggers lesion formation.^1^

Here, we describe a novel *GLMN* mutation in a patient presenting with congenital GVM and preaxial polydactyly—an unreported phenotypic association. Whole-exome sequencing (WES) did not reveal a secondary *GLMN* loss-of-function mutation but revealed a variant in cerebral cavernous malformations 2 protein-like (*CCM2L*), a gene involved in vascular development. This case expands the clinical spectrum of *GLMN*-associated anomalies and raises the possibility of genetic modifiers affecting disease expression.

## Case report

**Fig 1:**
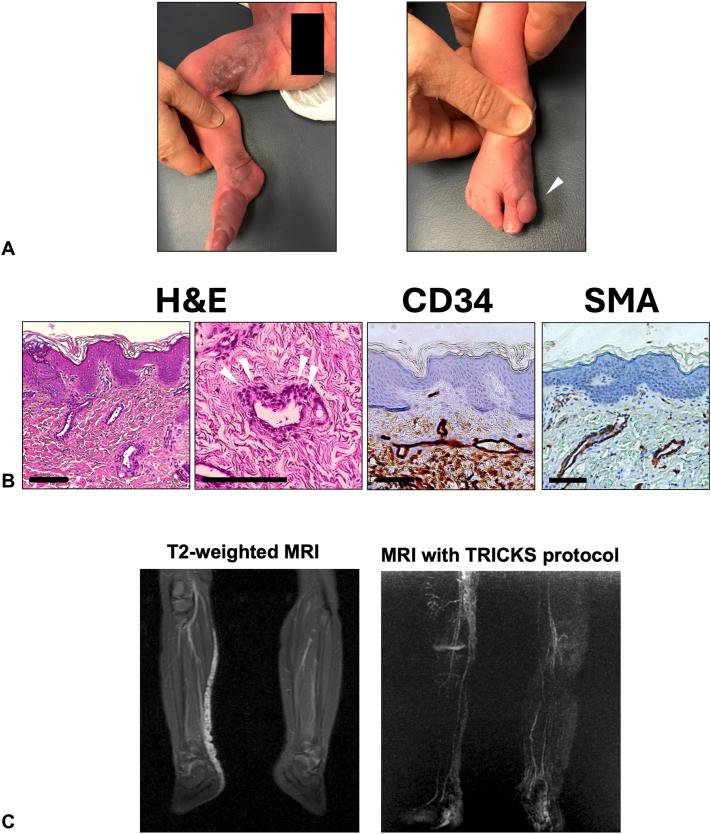
**A,** Clinical photographs of the right lower extremity. *Left*: A violaceous, firm plaque consistent with a GVM extends from the right anterior pelvic wall down to the distal great toe. *Right*: The lesion terminates distally in a supernumerary digit (*arrowhead*). **B,** Histopathology and immunohistochemistry of affected tissue. *Left 2 panels*: Hematoxylin and eosin (H&E) staining; glomus cells indicated by white arrowheads. *Middle*: CD34 immunostaining of endothelial cells. *Right*: Smooth muscle actin (SMA) staining of perivascular smooth muscle cells. Scale bars: 100 μm for panels 1 (H&E), 3 (CD34), and 4 (SMA); 130 μm for panel 2 (H&E). **C,** Radiological imaging of the lesion. *Left*: MRI showing T2 hyperintense vascular channels along the medial-posterior aspect of the right lower leg. *Right*: MRA using the TRICKS protocol reveals late-phase enhancement of the vascular lesion, consistent with a GVM. *GVM*, Glomuvenous malformation; *MRA*, magnetic resonance angiography; *MRI*, magnetic resonance imaging; *TRICKS*, time-resolved imaging of contrast kinetics.

A 2-week-old female patient presented with an extensive congenital violaceous plaque extending along the right leg and foot, terminating with a supernumerary digit on the medial aspect of the foot (Fig 1, *A*). She was born full-term via spontaneous vaginal delivery without complications, and there were no reported abnormalities during gestation. Histopathology revealed dilated CD34+ vessels lined by cuboidal cells expressing α-smooth muscle actin, consistent with a GVM (Fig 1, *B*). Magnetic resonance imaging showed enhancing plaque-like abnormalities in the skin and soft tissues extending from the anterior pelvic wall to the right foot, while magnetic resonance angiography with time-resolved imaging of contrast kinetics protocol demonstrated late-phase enhancement, both supporting the diagnosis (Fig 1, *C*).

The Blaschkoid distribution of the GVM raised the possibility of a postzygotic somatic mutation.^2^ We performed WES on genomic DNA from affected and unaffected skin. A *GLMN* c.515C>A, p.Ser172Ter mutation was identified at variant allele frequency ∼50% in both affected and unaffected samples, consistent with a heterozygous germline mutation. No secondary *GLMN* mutation was detected in lesional tissue. In the absence of a second-hit *GLMN* mutation, we explored other somatic variants using Mutect2 and Samtools.^3^^,^^4^ A rare c.1505G>A, p.Arg502His (R502H) disease-associated variant was identified in *CCM2L* with a variant allele frequency of 9% and combined annotation dependent depletion score 36, indicating a high likelihood of deleteriousness.^5^ The *CCM2L* mutation was detected exclusively in the disease tissue and was absent in the contralateral control DNA, consistent with a postzygotic somatic mutation.

**Fig 2:**
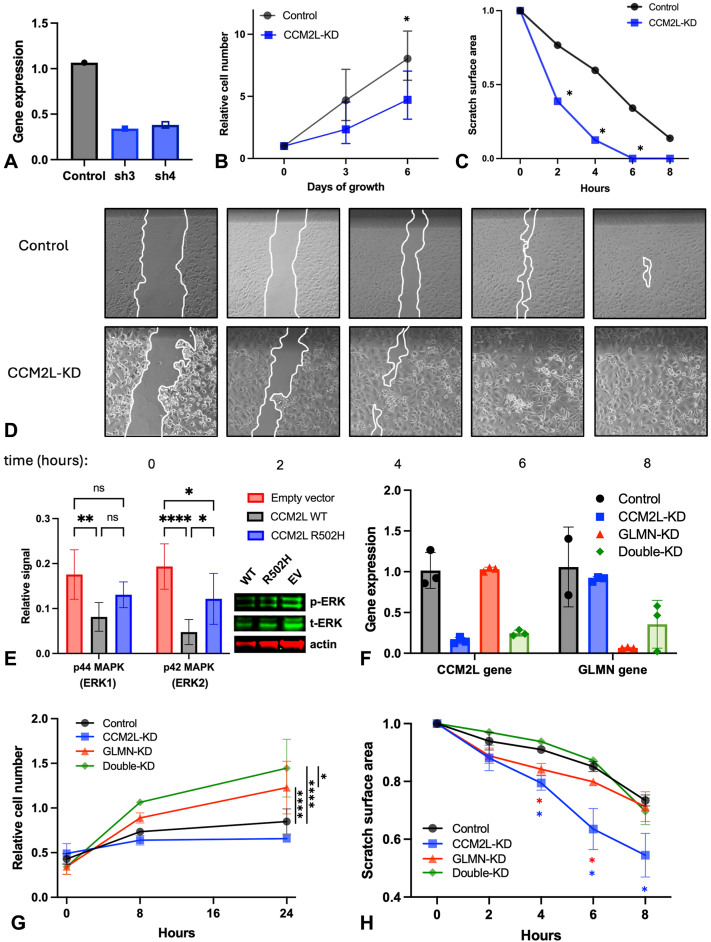
**A,** Quantitative PCR (qPCR) analysis of *CCM2L* expression following transduction with shRNA constructs, showing knockdown efficiency relative to control (ΔΔCT method). **B,** Cell proliferation assay of control and CCM2L knockdown cells measured over 6 days (2-way ANOVA, *P* = .0211). **C,** Quantification of scratch assay in control and CCM2L knockdown cells (2-tailed paired *t*-test, *P* = .0366). **D,** Representative time-course images from the scratch assay in control and CCM2L knockdown cells. **E,** Western blot analysis of phosphorylated ERK in HUVECs expressing wild-type or R502H CCM2L; quantification from 6 replicates (2-tailed unpaired *t*-test, *P* < .05, *n* = 6). **F,** qPCR analysis of GLMN, CCM2L, and combined knockdown efficiency following siRNA transfection (ΔΔCT method). **G,** Cell proliferation assay following siRNA-mediated knockdown of GLMN, CCM2L, or both, measured over time (2-way ANOVA with post hoc comparisons; ∗∗∗∗*P* < .0001, ∗*P* < .05). **H,** Wound closure assay in control, single, and double knockdown conditions (2-way repeated measures ANOVA, ∗*P* < .05). *ANOVA*, Analysis of variance; *CCM2L*, cerebral cavernous malformations 2 protein-like; *ERK*, extracellular signal-regulated kinase 1/2; *GLMN*, glomulin; *HUVECs*, human vascular endothelial cells; *PCR*, polymerase chain reaction.

*CCM2L*, implicated in cerebral cavernous malformations,^6^ regulates vessel stability and growth^7^ in part via mitogen-activated protein kinase/extracellular signal-regulated kinase 1/2 (ERK) signaling.^6^^,^^8^ However, its function in the skin has not been evaluated. We performed an experimental knockdown of CCM2L (Fig 2, *A*) in human vascular endothelial cells (HUVECs) and observed strong effects on cell proliferation and migration, with a 45% reduction in proliferation at 6 hours (2-way analysis of variance, *P* = .0211) and a 16% increase in migration speed compared to controls (2-tailed paired *t*-test, *P* = .0366) (Fig 2, *B*-*D*). Expression of wild-type versus R502H variant in HUVECs demonstrated that the mutated protein had an impaired ability to repress mitogen-activated protein kinase/ERK signaling, as evidenced by an increase in phosphorylated ERK levels in R502H compared to wild-type (Fig 2, *E*). To examine if combinatorial disruption of CCM2L and GLMN would have distinct cellular effects, we performed both individual and combinatorial knockdown (Fig 2, *F*). Double knockdown increased HUVEC proliferation more than GLMN single knockdown alone (Fig 2, *G*). By contrast, double knockdown did not alter HUVEC migration rate compared to control (Fig 2, *H*). We note that siRNA-mediated depletion may not precisely mimic the heterozygous loss-of-function observed clinically. These results suggested that *GLMN* and *CCM2L* have interacting and complex effects on HUVEC behavior and supported a biological function for the *CCM2L* c.1505G>A variant in vascular endothelial cells.

## Discussion

This study describes a novel GLMN c.515C>A, p.Ser172Ter mutation in a patient with congenital GVMs and preaxial polydactyly, expanding the phenotypic and genotypic spectrum of these vascular anomalies. GVMs are typically characterized by their congenital origin, extensive distribution, and progressive involvement after birth; however, they are usually associated with partial tissue atrophy rather than overgrowth. To our knowledge, this is the first reported case of polydactyly associated with GVM, suggesting an expanded phenotypic spectrum of *GLMN*-related vascular anomalies.

The 2-hit hypothesis for *GLMN* mutation-driven GVMs postulates a somatic second-hit event leading to biallelic inactivation.^9^ We did not detect a definitive secondary *GLMN* loss-of-function event in WES in the affected tissue, although we recognize that a localized second-hit mutation could fall below the detection threshold of WES of bulk tissue.^10^ We identified a rare *CCM2L* c.1505G>A, p.Arg502His variant in lesional tissue, suggesting it may act as a genetic modifier of lesion development. This variant is uncommon in the general population (A = 0.000002 in gnomAD), making it unlikely to be an incidental polymorphism. We found that CCM2L affects vascular cell proliferation, migration, and mitogen-activated protein kinase/ERK signaling, supporting a plausible biological function to modify vascular overgrowth in combination with GLMN. Our findings support future studies to further investigate whether *CCM2L* variants influence GVM severity or contribute to the spectrum of GLMN-related vascular disorders.

## Conflicts of interest

None disclosed.
